# Mode Effects on Self-Reported Health Across Web-Based, Interviewer-Administered, and Nurse-Administered Modes: Population-Based Study

**DOI:** 10.2196/89896

**Published:** 2026-10-16

**Authors:** Yunlong Liang, Tarek Al Baghal, Jonathan Burton, Meena Kumari

**Affiliations:** 1 Institute for Social and Economic Research, University of Essex, Wivenhoe Park, Colchester, Essex, CO4 3SQ, United Kingdom, 44 7703334101

**Keywords:** mode effects, surveys and questionnaires, computer-assisted personal interviewing, web-based surveys, social desirability bias, interviewer effects, mental health, population-based study

## Abstract

**Background:**

Probability-based surveys remain essential to epidemiology, but expanding mixed-mode data collection, which combines web self-completion and interviewer-administered surveys, raises concerns that administration mode and interviewer presence may influence health reporting. These concerns warrant further investigation of interviewer-related biases and mode differences in health reporting.

**Objective:**

This study assessed whether responses to the 12-item General Health Questionnaire (GHQ-12) and 12-item Short Form Health Survey (SF-12), both self-completed, differed by interviewer presence and role: online without an interviewer, during a social interviewer visit, or during a nurse visit. It also assessed mode effects on doctor-diagnosed conditions recorded by interviewers in computer-assisted personal interviewing (CAPI) and when self-completed online. We further examined possible variation by sex and age.

**Methods:**

The analysis used randomized mode allocation embedded within wave 12 (2019) of the UK Household Longitudinal Study Innovation Panel. After applying relevant exclusions, the final analytic sample comprised 1111 households (1779 participants) across the 3 randomly assigned survey modes: web-based self-completion, interviewer-administered CAPI, or nurse-administered CAPI. The sample was predominantly White British, with a broadly balanced sex distribution and participants from diverse age, employment, education, and household income groups. Participants did not always complete the survey in their randomly assigned mode. Effects were estimated using a two-stage residual inclusion instrumental variable approach, with randomized mode as an instrument for actual mode, to address confounding arising from nonrandom selection into actual mode.

**Results:**

No statistically significant differences were observed across self-completion contexts for the GHQ-12 or the SF-12 mental and physical component scores. We found no statistically significant evidence of mode effects on doctor-diagnosed conditions. For example, no statistically significant mode effects in psychiatric conditions were identified across the comparisons: nurse relative to social interviewer (odds ratio [OR] 0.72, 95% CI 0.40‐1.29; *P*=.53), web relative to social interviewer (OR 0.45, 95% CI 0.17‐1.18; *P*=.14), and web relative to nurse (OR 0.63, 95% CI 0.27‐1.49; *P*=.59). There was little evidence overall that mode-related differences varied by age or sex.

**Conclusions:**

Our findings may support embedding self-completion modules within interviewer-administered surveys for the collection of SF-12 and GHQ-12. Meanwhile, potential mode-related differences should be considered when designing mixed-mode surveys and interpreting self-reported health data.

## Introduction

Probability-based surveys have been a cornerstone of epidemiology and public health, providing reliable population-based estimates that enable the monitoring of population health trends to inform evidence-based policy [1]. Even amid the rise of alternative data sources, they remain essential to ensuring representativeness [2,3] and to capturing the social, psychological, behavioral, and biological determinants that shape health. Increasing emphasis on cost-efficiency and biomarker integration is accelerating the diversification of health survey administration. These modes include face-to-face interviews conducted by trained social interviewers, web-based self-administered surveys [4], and, where health is a focus, interviews conducted by nurses who can undertake standardized biospecimen collection and physical measurements [5]. The use of these different modes allows surveys to accommodate varying data-collection requirements.

However, the use of different data-collection modes within surveys may affect responses and introduce bias in health estimates [6]. Mode effects refer to systematic differences in survey responses that arise from the mode of data collection and its associated conditions [7]. Different mechanisms may underlie these effects. The presence of an interviewer may increase socially desirable responding, whereby respondents report in ways they perceive to be more socially acceptable [8], while interviewer probing, clarification, and feedback may also affect how respondents understand and report health conditions [9,10]. The former mechanism may be particularly relevant to sensitive or subjective scales. For example, previous studies with random assignment to survey mode found that interviewer-administered surveys yielded more favorable health ratings than self-administered surveys [11-15]. To reduce mode effects arising from social desirability bias, several national surveys embed self-completion within face-to-face computer-assisted personal interviewing (CAPI) for sensitive items [16-18]. However, the context in which self-completion responses are provided may differ across modes. Interviewer presence may shape reporting even when respondents complete items themselves by influencing perceived privacy, thereby contributing to differences between self-completion embedded within interviewer-present CAPI and web self-completion [10]. For example, one study found that respondents completing the 12-Item General Health Questionnaire (GHQ-12) in the presence of an interviewer reported better mental health than those completing it without an interviewer present [19]. Similarly, among self-administered modes, less interaction with interviewers was associated with higher reporting of common mental disorder symptoms [20]. However, these findings may reflect between-sample differences or evidence from specific populations, limiting their generalizability.

For the reporting of specific chronic conditions, however, studies have primarily compared interviewer-administered with self-administered reporting. Any observed differences in reporting may therefore reflect not only social desirability bias but also interviewer-specific features, such as probing and clarification. Several studies found higher prevalence estimates in self-administered surveys than in interviewer-administered surveys for a mental health problem [21], longstanding limiting illness, and current bodily pain lasting more than 6 months [22], possibly reflecting social desirability bias. One randomized study also found that most health problems were reported more frequently in a self-administered postal survey than in an interviewer-administered survey [23]. However, existing studies have been restricted to older adults or young people [21,23], with one of these studies additionally constrained by a small sample size [21], whereas one study was unable to separate mode differences from temporal differences [22]. Consequently, the extent to which survey mode independently affects the reporting of different individual health conditions in the broader adult population remains unclear. In contrast, interviewer probing and clarification may facilitate reporting of chronic conditions. For example, previous reports of higher prevalence of longstanding illness [9], bronchial asthma, and chronic bronchitis [24] in interviewer-administered surveys may reflect interviewer probing and clarification. However, other studies reported no mode differences in the number of chronic conditions, the prevalence of longstanding illness [19,25], or the prevalence of bronchial asthma and chronic bronchitis [9]. Differences in findings across studies may also reflect population selectivity, between-sample differences, or residual selection effects. Thus, evidence supporting interviewer facilitation as a mechanism underlying mode effects remains heterogeneous.

In addition, evidence suggests that interviewers’ professional roles can influence self-reported health [10], although the direction of these effects is inconsistent. Respondents interviewed by physicians or nurses have been reported to disclose fewer psychiatric symptoms [26] and fewer health problems than those interviewed by lay interviewers or completing self-administered questionnaires [23]. Conversely, higher pain intensity has been reported in some nurse-administered interviews [27]. However, these findings derive largely from specific subgroups or highly selected populations, limiting their generalizability. Meanwhile, whether interviewer effects associated with the professional role of nurses spill over into the self-administered modules embedded within CAPI surveys remains unclear. Furthermore, sex and age may modify the effects of survey mode on health reporting, but these differences have received limited attention. Sex-specific patterns may partly reflect variation in concerns about mental health stigma and disclosure. The greater perceived privacy of self-administered web surveys may facilitate the reporting of sensitive health information, particularly among men [21,28]. Age differences may reflect older adults’ stronger emphasis on socioemotional goals and greater reluctance to disclose negative states in interpersonal settings. Compared with less interpersonal modes, surveys involving greater interviewer contact have elicited fewer reports of adverse psychosocial functioning among older adults, with this difference becoming more pronounced with increasing age [29].

Therefore, this study aimed to examine whether health measures completed by respondents themselves are reported differently across contexts that differ in interviewer presence and roles, and whether the reporting of doctor-diagnosed chronic conditions differs between web self-completion, social interviewer-administered CAPI, and nurse-administered CAPI. We then examined whether these associations varied by sex and age groups in secondary analyses. Specifically, we asked the following questions:

- Although the GHQ-12 and SF-12 were self-completed in all 3 conditions, do participants report mental health and functioning differently across self-completion contexts involving no interviewer, a social interviewer, or a nurse?
- Does the reporting of diagnosed health conditions differ between web self-completion, social interviewer–administered CAPI, and nurse-administered CAPI?
- Do these associations differ by sex and age group?

## Methods

### Data

The UK Household Longitudinal Study Innovation Panel (UKHLS-IP) is a national probability sample that covers the household population of Great Britain, specifically designed to support methodological development and experimental research in longitudinal survey design [30]. The UKHLS-IP began in 2008, with a sampling design that used a stratified, geographically clustered probability approach yielding 2760 addresses [31], and was refreshed at waves 4 (2011), 7 (2014), 10 (2017), and 11 (2018) [32]

The Innovation Panel wave 12 (IP12) issued sample comprised 2401 households: 1957 households participated as recontacts from IP11, and 444 returning households from earlier waves. Before fieldwork, these 2401 households were randomly assigned to one of three modes: (1) web-based self-completion, (2) face-to-face CAPI by social interviewers, or (3) nurse-led in-home CAPI [33]. The sample flow is described in the *Results* section.

### Outcomes

The following health outcomes were chosen. Mental health was assessed using the GHQ-12 and SF-12 Mental Component Summary (MCS) scores. In all modes, respondents self-completed the scales, either as computer-assisted self-interviewing (CASI) modules within social-interviewer or nurse CAPI visits, or independently online. During the CAPI interview, the script instructed the social interviewer or nurse to pass the laptop to the respondent at the CASI section, which the respondent then completed independently. The interviewer or nurse generally remained in the room but attended to their own tasks and did not administer the questions or interact with the respondent during CASI completion, except when assistance was needed because of sight, reading, or language difficulties.

Each item in the GHQ-12 was rated on a 4-point Likert scale, yielding a total GHQ-12 score ranging from 0 (“no psychological distress”) to 36 (“severe psychological distress”), with higher scores indicating greater levels of distress [34]. The Physical Component Summary (PCS) score from the SF-12 was used to assess physical health. SF-12 MCS and SF-12 PCS scores were computed using the norm-based scoring algorithm [35]. Both scores were derived by the UKHLS team. We reverse-coded the scores such that higher values represented poorer physical and mental functioning, which allowed for effective interpretation and comparability.

In addition, we derived 6 binary indicators to capture major categories of chronic conditions, based on self-reported doctor diagnoses at IP12. Respondents were shown a list of specific conditions, on a showcard in the face-to-face modes and on screen in the web mode, and asked whether a doctor or other health professional had ever told them that they had any of these conditions. The measures, therefore, reflected doctor-diagnosed conditions reported up to the IP12 interview. These questions were ordinarily asked at a respondent’s first full individual interview. At IP12, however, both new entrants to the survey and continuing respondents were asked these questions. To improve statistical reliability and mitigate data sparsity, chronic conditions were grouped into six categories: (1) cancer, defined as any malignant neoplasm; (2) cardiovascular conditions, including congestive heart failure, coronary heart disease, angina, myocardial infarction, stroke, or high blood pressure; (3) metabolic–endocrine disorders, comprising diabetes or hypothyroidism; (4) musculoskeletal disorders, including arthritis; (5) psychiatric conditions, indicating whether respondents reported any doctor-diagnosed psychiatric disorder; and (6) respiratory conditions, including asthma, emphysema, chronic bronchitis, or chronic obstructive pulmonary disease. For each category, a value of 1 was assigned if any relevant condition was reported, and zero otherwise. The specific component conditions included in each major category, along with the number of participants reporting each condition, were described in Table S1 in Multimedia Appendix 1.

### Survey Mode

The primary exposure was the actual survey mode completed by each respondent, categorized as web-based self-completion, interviewer-administered, or nurse-administered interviews. For the GHQ-12 and SF-12, all items were self-completed by respondents, including within the CASI module of CAPI interviews; for these outcomes, the exposure therefore represents the context of self-completion, namely online without an interviewer present, during a social interviewer visit, or during a nurse visit, rather than interviewer administration of the items. For doctor-diagnosed chronic conditions, the exposure reflects both survey mode and item administration, as conditions were self-reported in the web questionnaire but were elicited using a condition list in the CAPI interviews.

### Covariates

Based on prior studies, we adjusted for a comprehensive set of contextual, sociodemographic, and survey-related variables to account for potential confounding [36-38]. Sex was coded as male or female. Gender identity and intersex variations were not available in the analytic data. Age was categorized into 6 groups: 16‐25, 26‐35, 36‐45, 46‐55, 56‐65, and ≥66 years. However, because the prevalence of chronic conditions was very low at younger ages and increased sharply from the mid-50s onward, we used a binary age indicator (<56 vs ≥56 y) in models with chronic disease as the outcome to avoid sparse-data bias and to ensure sufficient events within each category for stable estimation. Ethnicity was classified as White British and other ethnicities. Marital status was grouped into married, never married, and separated/divorced/widowed. Employment status was categorized as employed (including self-employed, unpaid family workers, and apprentices), unemployed and inactive (including students, individuals unable to work due to health conditions/disabilities, and family caregivers), and retired [39]. Education was grouped into degree or equivalent, General Certificate of Secondary Education (GCSE) and A-levels or equivalent, and no qualifications or other. Monthly household income was divided into quintiles: (0‐1969.13], (1969.13‐2956.40], (2956.40‐4083.32], (4083.32‐5756.04], and (5756.04‐20,000]. Household child status was based on the number of children, coded as none and ≥1 child.

The first participation wave was indicated by the wave of entry into the study: original sample in 2008, and refreshment samples in 2011, 2014, 2017, and 2018. Interview mode in IP11 was classified into 3 categories: interviewer-administered, web self-completion, and absence or proxy. Due to the small number of proxy respondents (n=12), they were combined with the absence of respondents.

Geographic regions included England, Wales, and Scotland. Urban-rural classification was based on population density, categorized as urban or rural areas.

### Statistical Analysis

For descriptive analyses, we summarized sample characteristics using survey-design–weighted means with 95% CIs for continuous variables and weighted proportions with 95% CIs for categorical variables, based on the imputed dataset. All summaries were stratified by survey mode.

For the main analyses, we used a two-stage residual inclusion (2SRI) approach within the instrumental variable framework, with the initial random assignment used as the instrument for the survey mode actually completed [40-42]. To be valid, instrumental variables must satisfy 4 identification assumptions: relevance, independence, exclusion restriction, and monotonicity (Table 1; more details can be found in Multimedia Appendix 1). We used this approach because some respondents did not complete the survey in their assigned mode; consequently, the mode actually completed was not entirely random and may have been subject to selection. Additionally, analyzing by initial assignment preserves randomization but can be diluted by mode switching, while analyses based on the completed mode could be confounded by differences between those who did and did not switch modes. The instrumental variable approach may reduce these concerns by using the difference in completed mode that was created by randomization. Because that difference originates from randomization rather than from participants’ own choices, it provides a basis for estimating the effect of completing that mode. Noncompliance and crossover are therefore incorporated through the extent to which assignment actually changed mode completion.

**Table 1.** Summary of instrumental variable assumptions and assessment.

Assumption	Assumption description	Assumption assessment	Conclusion and handling
Instrument relevance	Random assignment should be strongly associated with the survey mode actually completed.	First-stage models showed strong associations between assigned mode and completed mode. Detailed estimates and tests are reported in the Multimedia Appendix 1.	This assumption was satisfied.
Conditional independence	Random assignment should be independent of factors that may affect the outcomes, after accounting for measured covariates.	Assignment was randomized before fieldwork. Moderate covariate imbalances were observed (Table S2 in Multimedia Appendix 1), but the asymmetric switching rules operated only postrandomization and therefore do not undermine the initial assignment mechanism.	We used a conditional version of this assumption and adjusted for all prespecified covariates in both stages of the model.
Exclusion restriction	Random assignment should affect outcomes only through the survey mode actually completed.	Random assignment occurred before respondents had any survey exposure, making a direct effect of assignment on self-reported health through pathways other than the exposure unlikely.	This assumption was considered plausible, although it cannot be directly tested. Covariate adjustment was used to reduce potential alternative pathways.
Monotonicity	Assignment to a given mode should not reduce the probability of completing the interview in that mode.	Observed asymmetric switching does not, by itself, violate monotonicity. Defier status depends on treatment uptake under both assignment states and therefore cannot be inferred from a one-sided switch. Moreover, nurse-to-web switching is not evidence of defiance when nurse administration was unavailable under the comparison assignments.	This assumption was considered plausible but is not directly testable at the individual level.

In the first stage, we fitted a multinomial logistic regression model to predict respondents’ actual survey completion modes, using randomized mode assignment as the instrumental variable. The first-stage model estimated an allocation arm-specific vector of probabilities for the three actual completion modes. The model adjusted for a set of covariates, including demographic characteristics, household composition, socioeconomic position, first participation wave, interview mode in IP11, and geographic indicators. By design, nurse completion did not occur among participants allocated to the web or social interviewer arms, and social interviewer completion did not occur in the nurse arm. Consistent with these switching restrictions, the fitted probabilities for mode transitions that did not occur were effectively zero. For each respondent, we then calculated mode-specific residuals as the difference between indicators of actual completion mode and the corresponding predicted probabilities from the first-stage model.

In the second stage, the residuals for the modes were included alongside the observed survey mode and covariates in regression models estimating the associations with key outcomes. These outcomes included the total score of GHQ-12, the PCS and MCS scores from the SF-12, and a set of binary indicators for health conditions (cancer, cardiovascular conditions, metabolic-endocrine conditions, musculoskeletal disorders, psychiatric conditions, and respiratory conditions). We fitted outcome‐specific regression models: Gaussian linear models for continuous outcomes (GHQ-12 score, SF-12 PCS, and MCS scores) and logistic regression models for each binary chronic condition. All previously described covariates were included in both stages of the 2SRI analysis [43]. To avoid overstating clinical relevance, we a priori adopted the lower bound of these ranges (PCS=1.8; MCS=1.5) as our threshold for clinical importance [44]. For the GHQ-12, where no consensus threshold exists, we adopted 0.58 points on the Likert-scored scale, based on prior work, as a conservative benchmark to distinguish trivial from nontrivial differences at the group level [45]. For the GHQ-12 and SF-12 outcomes, we additionally reported standardized effect sizes. For binary outcomes, we prespecified a minimum clinically important risk difference of 5 percentage points for each chronic condition.

To address missingness, we applied multiple imputation using a random forest approach. Compared to parametric approaches, random forests are capable of capturing complex nonlinear relationships and higher-order interactions, and exhibit greater robustness to outliers and model misspecification [46]. The imputation model included outcomes and all covariates used in both the main analysis and the instrumental variable model, ensuring congeniality [47]. In addition, given the complex multistage survey design, the sampling weights, primary sampling units and stratification variables were incorporated into the imputation to preserve the underlying survey structure and mitigate potential bias arising from neglecting design features [48]. In addition, we performed imputation separately within strata of the exposure, defined by the observed survey mode, allowing the covariate and outcome distributions and their associations to differ flexibly between exposure groups and reducing bias when exposure-confounder interactions are present [49]. A total of 50 iterations per imputation were run to facilitate convergence. Given that approximately 15% of the data were incomplete, we generated 15 imputed datasets (Table S3 in Multimedia Appendix 1).

In each dataset, a 2SRI model was independently estimated to quantify exposure effects. To account for the complex survey design, we specified a clustered and stratified design and incorporated survey weights. Design-based standard errors were used in all analyses. The weight compensates for unequal selection probabilities introduced at each sampling stage and for nonresponse in IP12, ensuring that the weighted respondent sample remains representative of the UK adult population living in households at the time of fieldwork [32]. The final estimates and SEs were pooled using Rubin’s rules to obtain overall effect estimates and CIs.

All statistical analyses were performed in RStudio. Two‐sided *P* values <.05 with a Bonferroni correction applied were considered statistically significant. Pairwise contrasts for the primary exposure (survey modes) and all interaction tests were adjusted for multiple comparisons using the Bonferroni method. The corresponding 95% CIs were Bonferroni-adjusted.

Interaction analyses were performed using a similar 2SRI approach in the imputed data accounting for complex survey design. The first-stage multinomial model for mode assignment was estimated in the full sample, after which residuals were included in second-stage models with survey-mode-by-sex and survey-mode-by-age-group interaction terms, respectively. To account for multiple comparisons, Bonferroni correction was applied across the subgroup-specific pairwise contrasts for each outcome.

As a supplementary analysis, we examined the total number of chronic-condition categories reported by each participant, ranging from 0 to 6. Because this outcome was a count variable, we used Poisson regression to compare the number of chronic-condition categories across survey modes, otherwise following the same modeling approach as the main chronic-condition analyses. Results are presented as incidence rate ratios (IRRs) with 95% CIs. As a sensitivity analysis, we repeated the main analyses after excluding participants whose completed survey mode differed from their originally allocated mode.

Additionally, we conducted a post hoc power analysis to assess the ability of the mode-specific sample sizes to detect a benchmark mode effect reported in previous literature for long-standing illness [9].

### Ethical Considerations

The UKHLS received ethical approval from the University of Essex Ethics Committee. For waves prior to IP12, ethical approval for the IP was granted as part of the approvals for the UKHLS main survey data collection. From IP12 onward, separate ethical approvals have been obtained for each wave of the IP. IP12 received approval from the National Health Service East of England-Essex Research Ethics Committee (reference 19/EE/0146). Additional ethical approval was not required for the secondary data analysis conducted in this study.

## Results

Following the fieldwork process, households were excluded due to noncontact, refusal, or other reasons, resulting in 1408 responding households: 442, 495, and 471 households in the social interviewer, web, and nurse groups, respectively. Further individual-level exclusions were then applied for refusals, proxy or incomplete interviews, incapacity, and children/adolescents. A small number of telephone-mode respondents were excluded from the analysis to avoid potential auditory-mode effects. In addition, respondents with a sampling weight of zero were excluded because such cases do not contribute to population estimates under this survey design [50]. The final analytic sample included 438 households (652 individuals) in the social interviewer group, 330 households (587 individuals) in the web group, and 343 households (540 individuals) in the nurse group, resulting in a sample of 1111 households and 1779 individuals. The sample flow diagram is provided in Figure 1.

**Figure 1.**
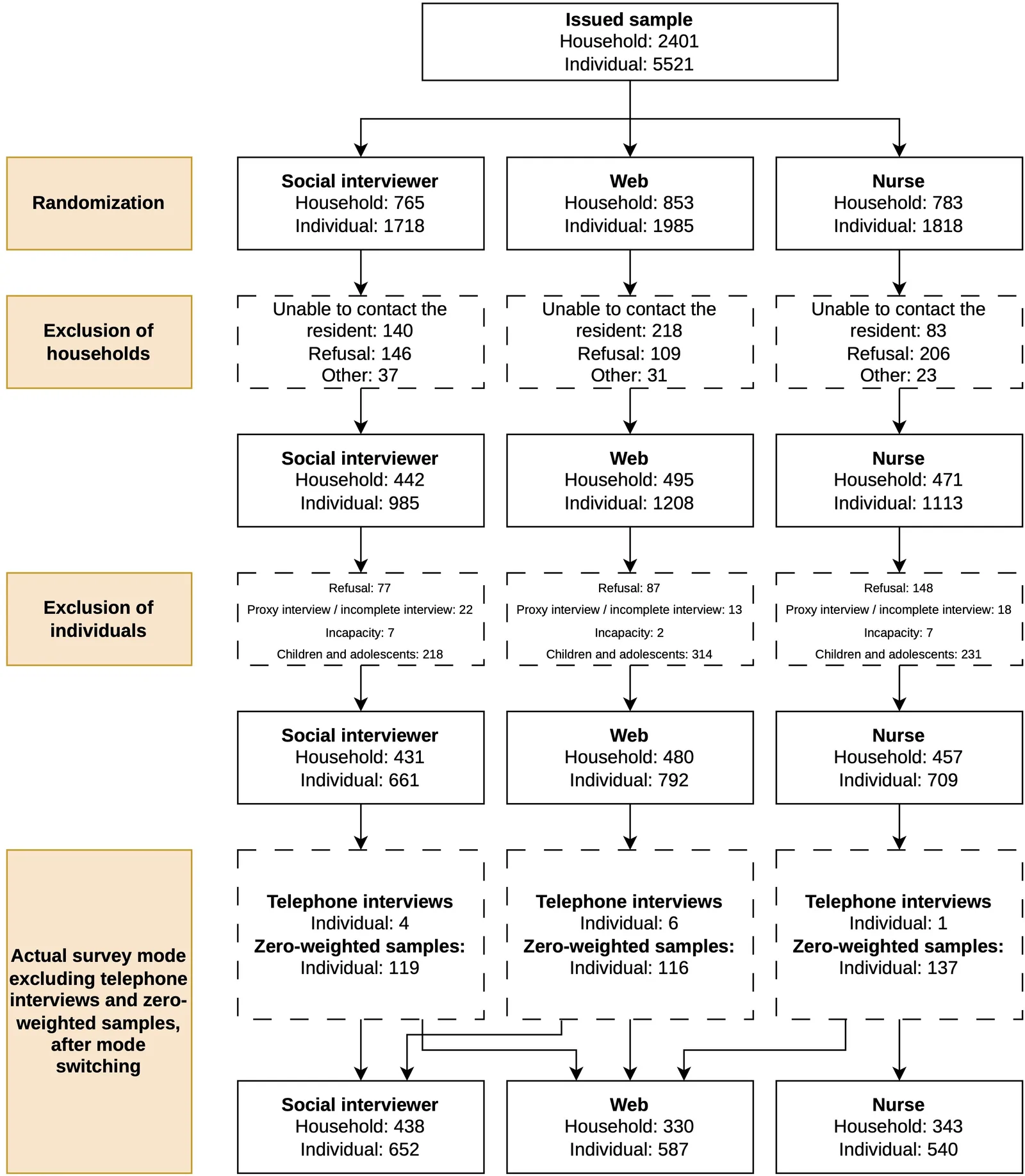
Flow diagram for the study sample.

Table S4 in Multimedia Appendix 1 summarizes sample characteristics from weighted imputed datasets by actual survey mode (n=1779). In the imputed, survey-weighted sample, respondents in the web-based self-completion group were physically healthier (SF-12 PCS score 22.12, 95% CI 21.32‐22.91) than those in either the interviewer-administered (SF-12 PCS score 26.54, 95% CI 25.44‐27.64) or nurse-administered (SF-12 PCS score 24.71, 95% CI 23.50‐25.91) CAPI groups. Web respondents also had a lower prevalence of cardiovascular conditions (unweighted 115/585; imputed, weighted 16.94%, 95% CI 13.21‐20.68) and respiratory conditions (63/585, 11.46%, 95% CI 7.83‐15.10) than did respondents in the social interviewer group (cardiovascular conditions: 187/651; 26.19%, 95% CI 22.41‐29.96; respiratory conditions: 124/651; 19.47%, 95% CI 15.67‐23.27), and fewer musculoskeletal disorders (74/585; 11.20%, 95% CI 8.37‐14.04) than nurse-administered respondents (102/540; 17.77%, 95% CI 14.16‐21.38).

Web respondents were younger, with more participants aged 16 to 25 years than in the social-interviewer group, and fewer aged 66 years or older. Compared with social-interviewer respondents, those in the web mode were generally more advantaged as they were less often separated, divorced, or widowed, less likely to have no formal qualifications, more likely to be employed, and less likely to be in the lowest income category. In addition, web respondents were significantly less likely to be separated, divorced, or widowed compared with respondents in the nurse mode. Web-mode respondents were less likely to have previously used interviewer-administered modes than respondents in the nurse mode or social-interviewer respondents, and were more likely to have previously used web self-completion.

Table 2 presents mode effect estimates for GHQ-12, the SF-12 MCS, and the SF-12 PCS, derived from a 2SRI approach that incorporates complex survey design features and multiple imputation for missingness. Across all mode comparisons, none of the estimated differences reached statistical significance. For GHQ-12, estimated differences ranged from −0.27 to 0.25 (effect sizes, −0.05 to 0.04; all *P*>.99); for SF-12 MCS, from −0.36 to 0.32 (effect sizes, −0.04 to 0.03; all *P*>.99); and for SF-12 PCS, from −1.02 to −0.36 (effect sizes, −0.10 to −0.03; *P*=.81 to >.99). Full pairwise estimates and 95% CIs are reported in Table 2. The findings of the complete case analyses were similar (Table S5 in Multimedia Appendix 1).

**Table 2.** Summary of results from the main analyses for 12-item General Health Questionnaire (GHQ-12), 12-item Short Form Mental Component Summary (SF-12 MCS), and 12-item Short Form Physical Component Summary (SF-12 PCS).

Outcomes	Estimate (95% CI)	Effect size	*P* value
GHQ-12			
Nurse (reference: social interviewer)	–0.27 (–1.38 to 0.84)	–0.05	>.99
Web (reference: nurse)	0.25 (–1.23 to 1.73)	0.04	>.99
Web (reference: social interviewer)	–0.02 (–1.97 to 1.94)	–0.01	>.99
SF-12 MCS			
Nurse (reference: social interviewer)	–0.36 (–2.14 to 1.42)	–0.04	>.99
Web (reference: nurse)	0.32 (–1.94 to 2.58)	0.03	>.99
Web (reference: social interviewer)	–0.04 (–2.99 to 2.91)	–0.01	>.99
SF-12 PCS			
Nurse (reference: social interviewer)	–0.36 (–2.25 to 1.53)	–0.03	>.99
Web (reference: nurse)	–0.66 (–2.20 to 0.88)	–0.06	.91
Web (reference: social interviewer)	–1.02 (–3.23 to 1.20)	–0.10	.81

Table 3 presents adjusted odds ratios (ORs; 95% CIs) from 2SRI logistic models that incorporate complex survey design and multiple imputation. For nurse vs social interviewer administration, ORs ranged from 0.72 to 1.56 across condition categories, with no consistent direction. In contrast, ORs for web vs nurse administration were consistently below 1, ranging from 0.54 to 0.90, corresponding to approximately 10% to 46% lower odds of reporting a doctor-diagnosed condition. Similarly, for web versus social interviewer administration, ORs were below 1 for 5 of the 6 condition categories (range 0.41‐0.86), corresponding to approximately 14% to 59% lower odds. Musculoskeletal disorders were the only exception to this pattern for web vs social interviewer administration (OR 1.15, 95% CI 0.52‐2.57). However, none of these pairwise comparisons reached statistical significance, and all 95% CIs included 1. The findings of the complete case analyses were similar (Table S6 in Multimedia Appendix 1), except for respiratory conditions when comparing respondents in the social interviewer (*P*=.005) mode with respondents in the web self-completion mode.

**Table 3.** Summary of results from the main analysis for cancer, cardiovascular conditions, metabolic-endocrine conditions, musculoskeletal disorders, psychiatric conditions, and respiratory conditions.

Outcomes	Odds ratio (95% CI)	*P* value
Cancer		
Nurse (reference: social interviewer)	1.23 (0.62-2.43)	>.99
Web (reference: nurse)	0.62 (0.24-1.63)	.72
Web (reference: social interviewer)	0.77 (0.25-2.35)	>.99
Cardiovascular conditions		
Nurse (reference: Social interviewer)	1.06 (0.65-1.71)	>.99
Web (reference: nurse)	0.64 (0.34-1.19)	.26
Web (reference: social interviewer)	0.68 (0.35-1.31)	.47
Metabolic-endocrine conditions		
Nurse (reference: social interviewer)	0.95 (0.52-1.75)	>.99
Web (reference: nurse)	0.90 (0.45-1.80)	>.99
Web (reference: social interviewer)	0.86 (0.36-2.04)	>.99
Musculoskeletal disorders		
Nurse (reference: social interviewer)	1.56 (0.89-2.72)	.17
Web (reference: nurse)	0.74 (0.43-1.27)	.54
Web (reference: social interviewer)	1.15 (0.52-2.57)	>.99
Psychiatric conditions		
Nurse (reference: social interviewer)	0.72 (0.40-1.29)	.53
Web (reference: nurse)	0.63 (0.27-1.49)	.59
Web (reference: social interviewer)	0.45 (0.17-1.18)	.14
Respiratory conditions		
Nurse (reference: social interviewer)	0.77 (0.44-1.36)	.82
Web (reference: nurse)	0.54 (0.27-1.09)	.11
Web (reference: social interviewer)	0.41 (0.17-1.03)	.06

Tables S7-S10 in Multimedia Appendix 1 present the results of moderation analyses stratified by sex and age group. There were no statistically significant differences in SF-12 or GHQ-12 scores between male and female individuals (Table S7 in Multimedia Appendix 1; overall interaction *P*>.99, except for GHQ-12, *P*=.25). The overall interaction between age group and survey mode was statistically significant for the SF-12 PCS score (*P*=.04) but none of the individual interaction contrasts reached statistical significance. The corresponding interactions were not statistically significant for the GHQ-12 or SF-12 MCS scores (Table S8 in Multimedia Appendix 1; *P*>.99).

For chronic conditions, there was no evidence of an overall interaction between sex and survey mode (all *P*>.99, except for musculoskeletal disorders, *P*=.25). Nevertheless, among men, musculoskeletal conditions were reported more frequently in nurse interviews than in social interviewer CAPI (OR 2.43, 95% CI 1.14‐5.20; *P*=.01) and in web self-completion mode (OR 0.43, 95% CI 0.19‐0.97; *P*=.04; reference: nurse mode; Table S9 in Multimedia Appendix 1). The corresponding risk differences exceeded 5% (Figure S1 in Multimedia Appendix 1). In contrast, among women, the effect estimates were closer to the null, with ORs of 1.15 (95% CI 0.52‐2.54) for nurse vs social interviewer, 1.10 (95% CI 0.51‐2.35) for web vs nurse, and 1.26 (95% CI 0.47‐3.41) for web vs social interviewer. For respiratory conditions, the direction of associations was consistent in men and women, with all pairwise ORs below 1, although the reductions were more pronounced among men. Among men, web self-completion was associated with lower odds than social interviewer CAPI (OR 0.32, 95% CI 0.10‐1.00; *P*=.049) and nurse CAPI (OR 0.45, 95% CI 0.20‐1.00; *P*=.05). Among women, the corresponding ORs were 0.53 and 0.64, respectively, with confidence intervals including 1. The overall interaction between age and survey modes was not statistically significant for any chronic condition (all *P*>.99, except for respiratory conditions, *P*=.86). Respiratory conditions were reported significantly less often via web-based self-completion among respondents aged 56 years and older compared to social interviewer CAPI (OR 0.25, 95% CI 0.09‐0.71; *P*=.003) and nurse CAPI (OR 0.32, 95% CI 0.13‐0.79; *P*=.005; Table S10 in Multimedia Appendix 1), with mode differences exceeding 5% (Figure S2 in Multimedia Appendix 1). However, the direction of associations was consistent across age groups; among respondents aged less than 56 years, the corresponding ORs for web versus social interviewer and web versus nurse were 0.53 and 0.70, respectively, although their confidence intervals included 1.

The results of the supplementary analysis of the number of chronic-condition categories are shown in Table S11 in Multimedia Appendix 1. Participants completing the survey via the web reported fewer chronic conditions than those assessed by nurses (IRR 0.72, 95% CI, 0.54‐0.96; *P*=.02). No significant differences were observed between nurse and social-interviewer modes (IRR 1.00, 95% CI, 0.83‐1.20; *P*>.99) or between web and social-interviewer modes (IRR 0.72, 95% CI, 0.50‐1.02; *P*=.07).

In sensitivity analyses excluding participants who completed a survey mode that differed from their originally allocated mode (Table S12 in Multimedia Appendix 1), findings were consistent with the main analyses. The only difference was that web mode (vs social interviewer) was associated with lower odds of respiratory conditions (OR 0.49; 95% CI, 0.26‐0.94; *P*=.03), whereas this association was marginal (*P*=.06) in the main analysis.

## Discussion

In this nationally representative study in Great Britain, we did not find clear evidence of differences in self-completed GHQ-12 or SF-12 scores across modes that varied in interviewer presence and role. We found no statistically significant evidence of mode-related differences in reports of doctor-diagnosed mental health and other conditions. However, the tendency for web self-completion to yield lower reporting of chronic conditions indicates that mode-related differences cannot be excluded. There was little evidence overall that mode-related differences varied by age or sex. Although some subgroup contrasts reached statistical significance, respiratory-disease estimates were broadly consistent across strata, while the musculoskeletal contrast within the male subgroup should be interpreted cautiously.

Although previous studies suggest that responses to self-completion instruments may vary according to the interview context [19,20], our study did not find clear evidence of differences in the self-completed GHQ-12 and the mental and physical components of the self-completed SF-12 across modes that varied in interviewer presence and role. Notably, a previous study found differences in GHQ-12 responses even though the GHQ-12 was self-completed by respondents in both interviewer-administered and self-completion survey modes, suggesting that contextual factors such as interviewer presence may still influence responses [19]. In contrast, the limited differences observed in our study may partly reflect the prior survey experience of the social interviewers and the study-specific briefing social interviewers and nurses received on fieldwork and interview procedures, together with CASI procedures that reduced direct interviewer involvement and protected the confidentiality of completed responses [51]. This interpretation is also consistent with recent evidence from a randomized study, which suggested that differences in the privacy and confidentiality afforded by self-completion modes may influence disclosure of mental health and other sensitive information [20]. Although our point estimates for GHQ-12 and SF-12 MCS were slightly more favorable in the nurse mode than in the social interviewer or web modes, the differences were negligible and not statistically significant. Differences between web and social interviewer modes were also minimal. These findings provide little evidence that either the presence of an interviewer during CAPI or the interviewer’s professional role introduced systematic bias in responses to the self-completed GHQ-12 or SF-12 instruments. Furthermore, our findings may support the use of appropriately implemented CASI within CAPI to minimize the mode effects previously observed when health scales were administered directly by interviewers [11,13,15,19,24]. This approach is widely adopted in many national surveys; for example, the 2023-2024 Adult Psychiatric Morbidity Survey in England similarly adopted a self-completion section within CAPI interviews to mitigate social desirability bias in sensitive item responses [18]. Overall, these findings may support the use of self-administered approaches, whether web-based or embedded as CASI within interviewer-administered surveys, particularly for administering sensitive health-related scales.

We also did not find statistically significant evidence of mode effects for doctor-diagnosed psychiatric conditions, similar to findings from previous research based on diagnostic algorithm measures [24]. A possible explanation is that factual questions with simple categorical response options may be less susceptible to social desirability bias than multi-item or more subjective mental health measures [9,24]. For other doctor-diagnosed chronic conditions, the reporting was also broadly similar across web self-completion and interviewer-administered modes, with no statistically significant evidence of mode effects. No consistent pattern emerged when comparing outcomes between nurse interviewers and lay interviewers. However, descriptive prevalence estimates tended to be lower for web self-completion, and adjusted OR estimates were generally in the direction of lower reporting for web self-completion, although the 95% CIs were wide and included the null. A supplementary analysis of the number of chronic-condition categories also suggested that web respondents reported fewer condition categories than those interviewed by nurses. The estimate was similar but less precise when web respondents were compared with social-interviewer respondents. This pattern is broadly consistent with previous research suggesting a tendency toward lower reporting of physical health conditions in web self-completion than in interviewer-administered modes [24]. One possible explanation is that interviewer probing and clarification facilitate the elicitation of chronic conditions that might otherwise go unreported in self-administered questionnaires [9]. Overall, although no statistically significant evidence of mode effects was observed for individual doctor-diagnosed chronic-condition categories, our findings provide further context for ongoing discussion of this potential mechanism. Mode-related differences in the overall reporting of doctor-diagnosed chronic conditions therefore cannot be excluded.

When the analysis was restricted to participants who did not undergo a mode switch, the association between web self-completion and respiratory conditions, relative to interviewer administration, strengthened from marginal to statistically significant (*P*=.03, compared with *P*=.06 in the main analysis), with respondents completing the survey online reporting fewer respiratory conditions. Previous evidence likewise suggests lower reporting of respiratory conditions in self-administered questionnaires [24], although findings across studies have not been consistent [9]. However, the overall pattern of point estimates for the other health conditions remained similar to that observed in the main analysis, with generally lower reported prevalence under web self-completion than interviewer administration, although these differences were not statistically significant. Thus, the finding for respiratory conditions may reflect a broader mode-related pattern in health reporting rather than an effect specific to respiratory conditions.

No significant overall interactions of self-completion contexts with age or sex were observed for GHQ-12 or SF-12, except for age differences in SF-12 PCS. However, as the age-stratified estimates were not statistically significant and direct supporting evidence is limited, this finding should be interpreted with caution. The overall interaction tests did not provide clear evidence that mode effects on chronic conditions varied by age or sex. Although individual contrasts reached statistical significance among adults aged 56 years or older and men, respiratory conditions showed a broadly consistent pattern across age and sex strata. This consistency suggests that the observed differences may reflect a general mode-related reporting pattern rather than clear effect modification by age or sex. Among men, musculoskeletal conditions were reported more frequently in the nurse-administered mode than in social interviewer or web self-completion modes, possibly because the nurse-administered context may facilitate reporting of symptoms that might otherwise be normalized or downplayed [52]. Given the nonsignificant overall interactions and the limited statistical power of the present study, the subgroup pattern should be regarded as exploratory.

This study offers several key advantages over prior research. First, we leveraged an existing randomized allocation of survey modes within a probability-based sample and applied an instrumental-variable approach to address potential selection bias in mode choice, thereby strengthening the generalizability and robustness of our results. Although our focus was on mode effects on measurement, we noted that self-selection can also operate through sample composition as a mode-related process. The instrumental-variable design helped mitigate concerns about such nonrandom mode choice. Second, inclusion of both standardized health scales and doctor-diagnosed chronic conditions allowed us to examine mode-related differences across different types of health indicators and administration conditions. Finally, by comparing the social interviewer-administered mode, web-based self-administration, and a nurse-administered mode, we offer additional insights into how nurse-administered CAPI may be associated with differences in self-reported health.

This study has several limitations. First, although our sample included participants from diverse racial and ethnic backgrounds, the small sample sizes of minority groups necessitated aggregation, limiting our ability to examine survey‐mode effects on self‐reported health outcomes within specific subgroups. Second, reports of doctor‐diagnosed chronic diseases were sparse and therefore combined by disease family, hindering further analysis of condition‐specific reporting patterns. Nevertheless, the effect estimates were generally in the direction of lower odds of reporting conditions with web self-completion than with interviewer-administered modes, but the 95% CIs were wide. Statistical power to detect mode-related differences was limited; post hoc power ranged from 57.2% to 61.1% across the 3 pairwise comparisons. Third, this study only assessed the association between survey mode and self‐reported health and did not directly assess disease-specific stigma. The study therefore could not assess whether the extent or direction of mode differences varied according to the perceived sensitivity or stigma associated with particular conditions. Finally, the asymmetric reallocation process may have resulted in residual confounding of the estimated effect of mode received. If unmeasured respondent characteristics such as internet access, literacy, health, or engagement with the study influenced which initially assigned mode was refused, the asymmetric reallocation structure could have differentially concentrated respondents with those characteristics in particular observed mode groups. Consequently, the estimated effect of mode received may partly reflect these asymmetry-induced compositional differences. However, sensitivity analyses restricted to participants who adhered to their original randomized mode allocation produced broadly similar findings, suggesting that the main results were unlikely to be primarily driven by the asymmetric reallocation of participants across survey modes. Moreover, the observed covariate imbalances between randomized groups indicate that the independence assumption cannot be regarded as fully satisfied on the basis of randomization alone. The instrumental variable approach therefore relied on conditional independence after adjustment for measured covariates. Residual confounding due to unmeasured factors cannot be excluded and remains a limitation.

Overall, this study provides important insights for the implementation of population-based health surveys. First, the absence of differences in GHQ-12 and SF-12 scores across self-completion contexts may support the use of CASI within face-to-face interviews for collecting these measures. Second, no statistically significant mode effects were observed for psychiatric or other doctor-diagnosed conditions. However, given the tendency for web self-completion to yield lower reporting of chronic conditions, mode-related differences, potentially reflecting interviewer probing and clarification, cannot be excluded and warrant further investigation. Finally, there was no clear evidence that mode-related reporting differed by age or sex; the musculoskeletal finding in men remains exploratory given limited power.

## Supplementary material

10.2196/89896 Multimedia Appendix 1 Supplementary materials.

## Abbreviations

2SRI: two-stage residual inclusion
CAPI: computer-assisted personal interviewing
CASI: computer-assisted self-interviewing
GCSE: General Certificate of Secondary Education
GHQ-12: 12-item General Health Questionnaire
IP: Innovation Panel
IRR: incidence rate ratio
MCS: Mental Component Summary
OR: odds ratio
PCS: Physical Component Summary
SF-12: 12-item Short Form Health Survey
UKHLS: UK Household Longitudinal Study
