# Supplementary material for: Mode Effects on Self-Reported Health Across Web-Based, Interviewer-Administered, and Nurse-Administered Modes: Population-Based Study
Source: JMIR Hum Factors. 2026 Oct 16;13:e89896. doi: 10.2196/89896 (PMC13674588; doi:10.2196/89896)
Supplement: Supplementary file 1 [file humanfactors-v13-e89896-s001.docx]

**Supplementary Methods: Instrumental Variable Assumptions and Diagnostics**

This supplementary section provides further details on the instrumental variable analysis. First, we estimated first-stage linear probability models for two endogenous treatment indicators, web and nurse (interviewer-administered CAPI as the reference), using the random assignment indicators as instruments and the same covariates as in the outcome equation, while accounting for the complex survey design. The joint Wald tests for the two assignment indicators produced values of F = 212.0 (P<.001) for the web versus social interviewer comparison and F = 5951.3 (P<.001) for nurse versus social interviewer, indicating strong instrument-treatment associations [1].

Second, the independence assumption requires that the instrument be as-if randomly assigned and unrelated to confounders that may affect the outcome. In our study, survey mode assignment was randomised before fieldwork, supporting this assumption theoretically. To empirically assess covariate balance across the randomisation groups, we accounted for the survey design and compared the distribution of covariates across the three groups (see Supplementary Table S2). We reported design-adjusted column percentages and absolute standardised mean differences (|SMD|). An |SMD| > 0.1 was generally considered indicative of imbalance [2]. Results showed that, apart from country of residence (|SMD| = 0.232), employment status, household income, age group, marital status, ethnicity, and first participation wave, exhibited moderate imbalance (0.10 ≤ |SMD| ≤ 0.25). To address imbalances and improve precision, we included all covariates in both the first and second stages of the 2SRI models, not just those with detected imbalance [3,4]. Thus, we adopted a weaker, conditional version of the assumption: that assignment was independent of both observed and unobserved confounders, conditional on the selected covariates.

Third, the exclusion restriction requires that the instrument affects the outcome only through the actual survey mode. Because assignment occurred prior to any survey exposure and respondents had not yet seen or completed any part of the questionnaire, it is reasonable to assume that the randomised assignment itself had no direct impact on respondents’ self-assessed health. Additionally, by including a set of covariates in the second-stage model, we reduced the scope for potential alternative pathways through which the instrument might be related to the outcome. These design features strengthen the plausibility of the exclusion restriction.

Fourth, we assumed mode-specific monotonicity. For each survey mode, assignment to that mode did not decrease, and may only increase, the probability of completing the interview in that mode relative to assignment to any other mode. Although monotonicity is untestable at the individual level [3], it is plausible here because modes were assigned centrally before any respondent contact, and assignment to one mode did not introduce additional deterrents relative to another. At the population level, this implied that the average probability of completion in that mode is higher under assignment to that mode than under assignment to any other mode.

**Reference**

1.	Baiocchi M, Cheng J, Small DS. Tutorial in Biostatistics: Instrumental Variable Methods for Causal Inference*. Stat Med 2014 Jun 15;33(13):2297–2340. PMID:24599889

2.	Austin PC. Balance diagnostics for comparing the distribution of baseline covariates between treatment groups in propensity-score matched samples. Stat Med 2009 Nov 10;28(25):3083–3107. PMID:19757444

3.	Walker V, Sanderson E, Levin MG, Damraurer SM, Feeney T, Davies NM. Reading and conducting instrumental variable studies: guide, glossary, and checklist. BMJ British Medical Journal Publishing Group; 2024 Oct 14;387. PMID:39401839

4.	Hemming K, Hall J, Copas A, Watson S, Taljaard M, Hooper R. Covariate adjustment in cluster randomised trials: a practical guide. BMJ British Medical Journal Publishing Group; 2025 Oct 24;391:e084194. PMID:41136249

**Table S1 Summary of self-reported diseases**

| **Disease Category** | **Disease** | **Reported Cases** |
| --- | --- | --- |
| **Cancer** | Cancer or malignancy | 97 |
| **Cardiovascular conditions** | Congestive heart failure | 16 |
|  | Coronary heart disease | 36 |
|  | Angina | 42 |
|  | Heart attack (myocardial infarction) | 47 |
|  | Stroke | 44 |
|  | Hypertension | 383 |
| **Metabolic-endocrine conditions** | Diabetes | 166 |
|  | Hypothyroidism | 79 |
| **Musculoskeletal disorders** | Arthritis | 287 |
| **Psychiatric conditions** | Psychiatric problem | 163 |
| **Respiratory conditions** | Asthma | 241 |
|  | Chronic bronchitis | 15 |
|  | Chronic obstructive pulmonary disease | 47 |
|  | Emphysema | 6 |

**Table S2 Covariate balance by randomised survey mode, accounted for the complex survey design**

| **Randomised allocation** | **Interviewer-administered CAPI** | **Web-based self-completion** | **Nurse-administered CAPI** | **test SMD** |
| --- | --- | --- | --- | --- |
| **Sex -** Female | 279.2 (51.1) | 282.7 (50.5) | 335.4 (49.9) | 0.016 |
| **Age** |  |  |  | **0.205** |
| 16-25 | 52.5 (9.6) | 75.6 (13.5) | 106.8 (15.9) |  |
| 26-35 | 82.4 (15.1) | 74.5 (13.3) | 108.3 (16.1) |  |
| 36-45 | 64.1 (11.7) | 91.9 (16.4) | 89.9 (13.4) |  |
| 46-55 | 86.5 (15.8) | 95.1 (17.0) | 109.2 (16.2) |  |
| 56-65 | 120.7 (22.1) | 86.2 (15.4) | 103.4 (15.4) |  |
| ≥66 | 140.4 (25.7) | 137.0 (24.5) | 154.6 (23.0) |  |
| **Ethnicity -** Other ethnicities | 33.9 ( 6.6) | 32.9 ( 6.2) | 77.8 (12.4) | **0.143** |
| **Marital status** |  |  |  | **0.157** |
| Married | 254.4 (47.6) | 250.3 (45.7) | 297.0 (45.2) |  |
| Never married | 175.7 (32.9) | 180.3 (32.9) | 269.0 (40.9) |  |
| Separated/Divorced/Widowed | 104.5 (19.5) | 117.4 (21.4) | 91.0 (13.9) |  |
| **Employment** |  |  |  | **0.106** |
| Employed | 287.8 (52.7) | 313.9 (56.3) | 356.4 (53.1) |  |
| Retired | 166.1 (30.4) | 152.1 (27.3) | 174.3 (26.0) |  |
| Unemployed & inactive | 92.6 (16.9) | 91.3 (16.4) | 140.1 (20.9) |  |
| **Education** |  |  |  | 0.082 |
| Degree or equivalent | 223.4 (41.8) | 227.9 (41.7) | 265.3 (40.7) |  |
| GCSE and A-levels or equivalent | 218.7 (41.0) | 246.3 (45.0) | 293.8 (45.0) |  |
| No qualifications or other | 91.9 (17.2) | 72.7 (13.3) | 93.2 (14.3) |  |
| **Family income per month** |  |  |  | **0.222** |
| (0,1969.128] | 135.1 (25.4) | 110.4 (20.8) | 144.9 (22.3) |  |
| (1969.128, 2956.395] | 116.0 (21.8) | 121.2 (22.9) | 117.4 (18.0) |  |
| (2956.395, 4083.321] | 114.9 (21.6) | 105.7 (19.9) | 116.1 (17.8) |  |
| (4083.321, 5756.043] | 98.2 (18.5) | 103.8 (19.6) | 114.9 (17.7) |  |
| (5756.043, 20000] | 67.4 (12.7) | 89.0 (16.8) | 157.4 (24.2) |  |
| **Children in household** - ≥ one children | 117.0 (22.0) | 147.5 (27.8) | 161.4 (24.8) | 0.090 |
| **Survey mode in wave 11** |  |  |  | 0.071 |
| CAPI | 284.8 (52.1) | 279.0 (49.8) | 315.4 (46.9) |  |
| Web-based self-completion | 217.8 (39.8) | 233.8 (41.7) | 292.7 (43.5) |  |
| Absence or proxy | 44.0 ( 8.0) | 47.5 ( 8.5) | 64.0 ( 9.5) |  |
| **First-wave participation** |  |  |  | **0.157** |
| IP original sample 2008 | 162.7 (29.8) | 151.0 (26.9) | 183.3 (27.3) |  |
| IP4 refreshment sample 2011 | 81.1 (14.8) | 87.8 (15.7) | 97.8 (14.5) |  |
| IP7 refreshment sample 2014 | 121.9 (22.3) | 106.9 (19.1) | 112.1 (16.7) |  |
| IP10 refreshment sample 2017 | 67.4 (12.3) | 66.1 (11.8) | 106.1 (15.8) |  |
| IP11 refreshment sample 2018 | 113.6 (20.8) | 148.5 (26.5) | 172.8 (25.7) |  |
| **Country** |  |  |  | **0.232** |
| England | 463.5 (85.4) | 496.0 (88.5) | 583.0 (86.7) |  |
| Scotland | 46.9 ( 8.6) | 52.3 ( 9.3) | 32.5 ( 4.8) |  |
| Wales | 32.5 ( 6.0) | 12.0 ( 2.1) | 56.6 ( 8.4) |  |
| **Urban or rural -** rural | 114.7 (21.1) | 106.2 (19.0) | 139.7 (20.8) | 0.036 |

**Table S3 Comparison of covariates between complete‑case data and 15 imputed datasets**

|  | **Unweighted profile - complete cases** | **Weighted profile - complete cases** | **Weighted profile - imputed** |
| --- | --- | --- | --- |
| GHQ-12 | 11.00 (10.75, 11.25) | 11.29 (10.90, 11.69) | 11.33 (10.95, 11.70) |
| SF-12 MCS | 29.42 (28.93, 29.91) | 30.33 (29.59, 31.06) | 30.40 (29.66, 31.14) |
| SF-12 PCS | 24.55 (24.04, 25.06) | 24.47 (23.80, 25.15) | 24.56 (23.89, 25.23) |
| Cancer |  |  |  |
| No | 94.18 (93.01, 95.35) | 95.12 (93.90, 96.34) | 95.51 (94.43, 96.60) |
| Yes | 5.82 (4.65, 6.99) | 4.88 (3.66, 6.10) | 4.49 (3.40, 5.57) |
| Cardiovascular conditions |  |  |  |
| No | 73.35 (71.15, 75.56) | 75.86 (72.93, 78.79) | 77.11 (74.40, 79.82) |
| Yes | 26.65 (24.44, 28.85) | 24.14 (21.21, 27.07) | 22.89 (20.18, 25.60) |
| Metabolic-endocrine conditions | |  |  |
| No | 85.71 (83.96, 87.45) | 86.73 (84.78, 88.68) | 87.75 (86.05, 89.46) |
| Yes | 14.29 (12.55, 16.04) | 13.27 (11.32, 15.22) | 12.25 (10.54, 13.95) |
| Musculoskeletal disorders |  |  |  |
| No | 83.25 (81.39, 85.11) | 84.46 (82.21, 86.72) | 85.26 (83.26, 87.26) |
| Yes | 16.75 (14.89, 18.61) | 15.54 (13.28, 17.79) | 14.74 (12.74, 16.74) |
| Psychiatric conditions |  |  |  |
| No | 90.82 (89.37, 92.26) | 89.32 (87.23, 91.41) | 89.63 (87.72, 91.54) |
| Yes | 9.18 (7.74, 10.63) | 10.68 (8.59, 12.77) | 10.37 (8.46, 12.28) |
| Respiratory conditions |  |  |  |
| No | 84.41 (82.60, 86.22) | 84.12 (81.94, 86.29) | 84.10 (81.96, 86.23) |
| Yes | 15.59 (13.78, 17.40) | 15.88 (13.71, 18.06) | 15.90 (13.77, 18.04) |
| Randomised allocation |  |  |  |
| Interviewer-administered CAPI | 30.72 (28.42, 33.02) | 31.09 (27.45, 34.73) | 30.72 (27.22, 34.23) |
| Nurse-administered CAPI | 31.89 (29.56, 34.21) | 31.41 (27.32, 35.50) | 31.50 (27.57, 35.42) |
| Web-based self-completion | 37.39 (34.97, 39.80) | 37.50 (33.74, 41.25) | 37.78 (34.13, 41.44) |
| Sex |  |  |  |
| Male | 45.86 (43.38, 48.34) | 50.03 (47.41, 52.65) | 49.56 (47.13, 51.99) |
| Female | 54.14 (51.66, 56.62) | 49.97 (47.35, 52.59) | 50.44 (48.01, 52.87) |
| Age |  |  |  |
| 16-25 | 7.76 (6.43, 9.10) | 11.39 (8.99, 13.78) | 13.21 (10.53, 15.89) |
| 26-35 | 10.22 (8.71, 11.73) | 13.89 (11.30, 16.49) | 14.90 (12.43, 17.37) |
| 36-45 | 13.52 (11.81, 15.22) | 14.30 (11.83, 16.77) | 13.82 (11.61, 16.03) |
| 46-55 | 17.40 (15.51, 19.29) | 16.18 (13.82, 18.54) | 16.35 (14.20, 18.50) |
| 56-65 | 19.15 (17.18, 21.11) | 17.99 (15.84, 20.15) | 17.44 (15.25, 19.63) |
| ≥66 | 31.95 (29.63, 34.28) | 26.25 (22.49, 30.01) | 24.28 (20.65, 27.92) |
| Ethnicity |  |  |  |
| White British | 94.37 (93.22, 95.52) | 92.75 (90.30, 95.21) | 91.45 (88.71, 94.19) |
| Other ethnicities | 5.63 (4.48, 6.78) | 7.25 (4.79, 9.70) | 8.55 (5.81, 11.29) |
| Marital status |  |  |  |
| Married | 55.17 (52.69, 57.65) | 46.05 (42.41, 49.69) | 45.76 (42.18, 49.34) |
| Never married | 25.61 (23.44, 27.79) | 35.17 (31.20, 39.14) | 36.46 (32.51, 40.41) |
| Separated/divorced/widowed | 19.21 (17.25, 21.18) | 18.78 (16.20, 21.36) | 17.79 (15.53, 20.04) |
| Employment status |  |  |  |
| Employed | 51.29 (48.80, 53.79) | 53.24 (49.29, 57.18) | 54.02 (50.12, 57.91) |
| Retired | 36.55 (34.14, 38.95) | 29.90 (25.98, 33.83) | 27.70 (23.81, 31.59) |
| Unemployed & inactive | 12.16 (10.53, 13.79) | 16.86 (14.17, 19.55) | 18.28 (15.61, 20.95) |
| Education |  |  |  |
| Degree or equivalent | 43.40 (40.93, 45.87) | 41.80 (39.06, 44.54) | 41.27 (38.34, 44.21) |
| GCSE and A-levels or equivalent | 41.98 (39.52, 44.44) | 42.77 (39.57, 45.97) | 43.82 (40.64, 46.99) |
| No qualifications or other | 14.62 (12.86, 16.38) | 15.43 (12.76, 18.10) | 14.91 (12.46, 17.36) |
| Monthly household income |  |  |  |
| (0,1969.128] | 20.38 (18.37, 22.38) | 23.02 (20.11, 25.93) | 22.47 (19.59, 25.35) |
| (1969.128, 2956.395] | 20.25 (18.24, 22.25) | 20.09 (17.52, 22.66) | 20.44 (17.57, 23.30) |
| (2956.395, 4083.321] | 20.25 (18.24, 22.25) | 20.39 (17.86, 22.93) | 19.50 (17.16, 21.83) |
| (4083.321, 5756.043] | 19.66 (17.68, 21.65) | 18.30 (15.58, 21.03) | 19.11 (16.40, 21.82) |
| (5756.043, 20000] | 19.47 (17.50, 21.44) | 18.20 (14.88, 21.51) | 18.49 (15.25, 21.72) |
| The number of children |  |  |  |
| None | 77.88 (75.81, 79.95) | 76.67 (73.33, 80.01) | 75.25 (71.76, 78.75) |
| >= one children | 22.12 (20.05, 24.19) | 23.33 (19.99, 26.67) | 24.75 (21.25, 28.24) |
| Interview mode in Wave 11 |  |  |  |
| Interviewer-administered | 50.19 (47.70, 52.69) | 52.20 (48.36, 56.03) | 49.42 (45.70, 53.14) |
| Web self-completion | 44.18 (41.70, 46.65) | 41.13 (37.33, 44.94) | 41.84 (38.20, 45.47) |
| Absence or proxy | 5.63 (4.48, 6.78) | 6.67 (4.83, 8.52) | 8.74 (6.79, 10.69) |
| First-wave participation |  |  |  |
| IP original sample 2008 | 29.88 (27.60, 32.17) | 25.86 (22.39, 29.34) | 27.94 (24.47, 31.40) |
| IP4 refreshment sample 2011 | 15.72 (13.90, 17.53) | 15.27 (12.38, 18.17) | 14.99 (12.17, 17.81) |
| IP7 refreshment sample 2014 | 18.89 (16.94, 20.84) | 20.11 (16.66, 23.56) | 19.16 (15.92, 22.41) |
| IP10 refreshment sample 2017 | 12.23 (10.59, 13.86) | 14.03 (11.15, 16.90) | 13.47 (10.78, 16.15) |
| IP11 refreshment sample 2018 | 23.29 (21.18, 25.39) | 24.73 (21.65, 27.81) | 24.44 (21.16, 27.73) |
| Country |  |  |  |
| England | 88.49 (86.89, 90.08) | 85.70 (80.93, 90.48) | 86.92 (82.76, 91.07) |
| Scotland | 6.14 (4.95, 7.34) | 7.87 (5.52, 10.22) | 7.41 (5.07, 9.74) |
| Wales | 5.37 (4.24, 6.49) | 6.42 (1.58, 11.26) | 5.68 (1.55, 9.81) |
| Urban or rural |  |  |  |
| Urban area | 25.42 (23.25, 27.59) | 20.72 (14.60, 26.83) | 20.31 (14.36, 26.25) |
| Rural area | 74.58 (72.41, 76.75) | 79.28 (73.17, 85.40) | 79.69 (73.75, 85.64) |

**Table S4 Characteristics of the study sample, overall and by actual survey modes**

|  | **Weighted profile - imputed** | | **Weighted profile - imputed: Social interviewer** | | **Weighted profile - imputed: Web** | | **Weighted profile - imputed: Nurse** |
| --- | --- | --- | --- | --- | --- | --- | --- |
| GHQ-12 | 11.33 (10.95, 11.70) | | 11.65 (11.02, 12.27) | | 11.24 (10.39, 12.10) | | 11.01 (10.40, 11.61) |
| SF-12 MCS | 30.40 (29.66, 31.14) | | 30.50 (29.33, 31.68) | | 30.69 (29.43, 31.94) | | 29.95 (28.90, 31.01) |
| SF-12 PCS | 24.56 (23.89, 25.23) | | 26.54 (25.44, 27.64) | | 22.12 (21.32, 22.91) | | 24.71 (23.50, 25.91) |
| Cancer |  | |  | |  | |  |
| No | 95.51 (94.43, 96.60) | | 94.84 (93.07, 96.61) | | 96.91 (95.67, 98.14) | | 94.85 (92.78, 96.92) |
| Yes | 4.49 (3.40, 5.57) | | 5.16 (3.39, 6.93) | | 3.09 (1.86, 4.33) | | 5.15 (3.08, 7.22) |
| Cardiovascular conditions |  | |  | |  | |  |
| No | 77.11 (74.40, 79.82) | | 73.81 (70.04, 77.59) | | 83.06 (79.32, 86.79) | | 74.85 (69.68, 80.03) |
| Yes | 22.89 (20.18, 25.60) | | 26.19 (22.41, 29.96) | | 16.94 (13.21, 20.68) | | 25.15 (19.97, 30.32) |
| Metabolic-endocrine conditions | |  | |  | |  |  |
| No | 87.75 (86.05, 89.46) | | 86.43 (83.67, 89.18) | | 90.26 (87.57, 92.96) | | 86.72 (83.03, 90.40) |
| Yes | 12.25 (10.54, 13.95) | | 13.57 (10.82, 16.33) | | 9.74 (7.04, 12.43) | | 13.28 (9.60, 16.97) |
| Musculoskeletal disorders |  | |  | |  | |  |
| No | 85.26 (83.26, 87.26) | | 84.63 (81.22, 88.05) | | 88.80 (85.96, 91.63) | | 82.23 (78.62, 85.84) |
| Yes | 14.74 (12.74, 16.74) | | 15.37 (11.95, 18.78) | | 11.20 (8.37, 14.04) | | 17.77 (14.16, 21.38) |
| Psychiatric conditions |  | |  | |  | |  |
| No | 89.63 (87.72, 91.54) | | 87.42 (83.82, 91.03) | | 91.69 (88.81, 94.57) | | 90.19 (87.72, 92.66) |
| Yes | 10.37 (8.46, 12.28) | | 12.58 (8.97, 16.18) | | 8.31 (5.43, 11.19) | | 9.81 (7.34, 12.28) |
| Respiratory conditions |  | |  | |  | |  |
| No | 84.10 (81.96, 86.23) | | 80.53 (76.73, 84.33) | | 88.54 (84.90, 92.17) | | 83.82 (80.08, 87.57) |
| Yes | 15.90 (13.77, 18.04) | | 19.47 (15.67, 23.27) | | 11.46 (7.83, 15.10) | | 16.18 (12.43, 19.92) |
| Randomised allocation |  | |  | |  | |  |
| Interviewer-administered CAPI | 30.72 (27.22, 34.23) | | 69.93 (63.85, 76.00) | | 13.17 (7.80, 18.54) | | 0.00 (0.00, 0.00) |
| Nurse-administered CAPI | 31.50 (27.57, 35.42) | | 0.00 (0.00, 0.00) | | 5.14 (2.48, 7.81) | | 100.00 (100.00, 100.00) |
| Web-based self-completion | 37.78 (34.13, 41.44) | | 30.07 (24.00, 36.15) | | 81.69 (75.87, 87.50) | | 0.00 (0.00, 0.00) |
| Sex |  | |  | |  | |  |
| Male | 49.56 (47.13, 51.99) | | 49.23 (45.36, 53.10) | | 50.24 (45.87, 54.61) | | 49.25 (45.23, 53.26) |
| Female | 50.44 (48.01, 52.87) | | 50.77 (46.90, 54.64) | | 49.76 (45.39, 54.13) | | 50.75 (46.74, 54.77) |
| Age |  | |  | |  | |  |
| 16-25 | 13.21 (10.53, 15.89) | | 8.75 (5.94, 11.57) | | 18.46 (13.32, 23.60) | | 13.17 (8.30, 18.04) |
| 26-35 | 14.90 (12.43, 17.37) | | 15.60 (11.61, 19.59) | | 16.71 (12.03, 21.39) | | 12.06 (8.26, 15.87) |
| 36-45 | 13.82 (11.61, 16.03) | | 11.77 (8.36, 15.17) | | 13.34 (9.70, 16.98) | | 16.95 (13.07, 20.82) |
| 46-55 | 16.35 (14.20, 18.50) | | 14.36 (11.20, 17.51) | | 18.13 (14.33, 21.94) | | 16.94 (12.31, 21.57) |
| 56-65 | 17.44 (15.25, 19.63) | | 20.97 (16.95, 24.99) | | 15.16 (11.67, 18.64) | | 15.43 (11.62, 19.24) |
| ≥66 | 24.28 (20.65, 27.92) | | 28.56 (23.80, 33.32) | | 18.20 (13.54, 22.86) | | 25.45 (20.45, 30.45) |
| Ethnicity |  | |  | |  | |  |
| White British | 91.45 (88.71, 94.19) | | 91.36 (86.35, 96.37) | | 90.69 (86.33, 95.04) | | 92.40 (88.81, 96.00) |
| Other ethnicities | 8.55 (5.81, 11.29) | | 8.64 (3.63, 13.65) | | 9.31 (4.96, 13.67) | | 7.60 (4.00, 11.19) |
| Marital status |  | |  | |  | |  |
| Married | 45.76 (42.18, 49.34) | | 42.86 (37.05, 48.67) | | 48.81 (42.83, 54.80) | | 46.13 (40.04, 52.21) |
| Never married | 36.46 (32.51, 40.41) | | 35.48 (30.34, 40.61) | | 41.81 (36.16, 47.45) | | 31.90 (25.76, 38.04) |
| Separated/divorced/widowed | 17.79 (15.53, 20.04) | | 21.66 (17.63, 25.69) | | 9.38 (6.68, 12.08) | | 21.97 (17.21, 26.74) |
| Employment status |  | |  | |  | |  |
| Employed | 54.02 (50.12, 57.91) | | 47.82 (42.95, 52.69) | | 59.29 (53.16, 65.42) | | 56.17 (51.15, 61.18) |
| Retired | 27.70 (23.81, 31.59) | | 31.13 (25.73, 36.52) | | 23.25 (17.64, 28.86) | | 28.18 (23.18, 33.19) |
| Unemployed & inactive | 18.28 (15.61, 20.95) | | 21.05 (16.41, 25.69) | | 17.46 (13.74, 21.18) | | 15.65 (11.07, 20.23) |
| Education |  | |  | |  | |  |
| Degree or equivalent | 41.27 (38.34, 44.21) | | 37.10 (32.27, 41.93) | | 45.44 (40.69, 50.18) | | 42.07 (36.67, 47.46) |
| GCSE and A-levels or equivalent | 43.82 (40.64, 46.99) | | 41.88 (37.41, 46.36) | | 45.71 (40.91, 50.51) | | 44.22 (38.70, 49.74) |
| No qualifications or other | 14.91 (12.46, 17.36) | | 21.02 (16.86, 25.18) | | 8.85 (6.00, 11.71) | | 13.72 (9.43, 18.01) |
| Monthly household income |  | |  | |  | |  |
| (0,1969.128] | 22.47 (19.59, 25.35) | | 29.17 (24.07, 34.26) | | 15.97 (12.12, 19.82) | | 21.03 (16.68, 25.38) |
| (1969.128, 2956.395] | 20.44 (17.57, 23.30) | | 21.98 (17.50, 26.46) | | 16.48 (11.64, 21.33) | | 22.76 (16.96, 28.56) |
| (2956.395, 4083.321] | 19.50 (17.16, 21.83) | | 18.02 (13.35, 22.70) | | 20.85 (16.47, 25.23) | | 19.90 (14.30, 25.50) |
| (4083.321, 5756.043] | 19.11 (16.40, 21.82) | | 17.59 (13.44, 21.74) | | 20.51 (15.58, 25.44) | | 19.51 (13.76, 25.26) |
| (5756.043, 20000] | 18.49 (15.25, 21.72) | | 13.24 (8.96, 17.51) | | 26.19 (19.67, 32.71) | | 16.81 (11.75, 21.86) |
| The number of children |  | |  | |  | |  |
| None | 75.25 (71.76, 78.75) | | 77.40 (71.53, 83.27) | | 75.76 (70.72, 80.80) | | 71.99 (65.85, 78.12) |
| >= one children | 24.75 (21.25, 28.24) | | 22.60 (16.73, 28.47) | | 24.24 (19.20, 29.28) | | 28.01 (21.88, 34.15) |
| Interview mode in Wave 11 |  | |  | |  | |  |
| Interviewer-administered | 49.42 (45.70, 53.14) | | 64.12 (58.17, 70.06) | | 29.67 (25.23, 34.10) | | 52.17 (44.42, 59.92) |
| Web self-completion | 41.84 (38.20, 45.47) | | 27.94 (22.94, 32.95) | | 60.29 (55.19, 65.39) | | 39.48 (32.22, 46.73) |
| Absence or proxy | 8.74 (6.79, 10.69) | | 7.94 (4.47, 11.41) | | 10.05 (7.09, 13.00) | | 8.35 (4.92, 11.78) |
| First-wave participation |  | |  | |  | |  |
| IP original sample 2008 | 27.94 (24.47, 31.40) | | 29.07 (23.47, 34.68) | | 27.12 (21.76, 32.47) | | 27.38 (20.62, 34.14) |
| IP4 refreshment sample 2011 | 14.99 (12.17, 17.81) | | 14.72 (10.74, 18.69) | | 16.22 (11.96, 20.49) | | 14.01 (8.92, 19.09) |
| IP7 refreshment sample 2014 | 19.16 (15.92, 22.41) | | 18.25 (12.98, 23.52) | | 19.78 (14.49, 25.07) | | 19.65 (12.91, 26.39) |
| IP10 refreshment sample 2017 | 13.47 (10.78, 16.15) | | 14.71 (10.29, 19.12) | | 13.10 (7.77, 18.43) | | 12.29 (8.68, 15.90) |
| IP11 refreshment sample 2018 | 24.44 (21.16, 27.73) | | 23.26 (18.78, 27.74) | | 23.77 (17.85, 29.70) | | 26.67 (20.44, 32.91) |
| Country |  | |  | |  | |  |
| England | 86.92 (82.76, 91.07) | | 83.77 (78.00, 89.55) | | 89.37 (83.54, 95.20) | | 88.25 (82.69, 93.81) |
| Scotland | 7.41 (5.07, 9.74) | | 7.28 (4.49, 10.06) | | 5.45 (2.74, 8.17) | | 9.69 (4.08, 15.29) |
| Wales | 5.68 (1.55, 9.81) | | 8.95 (3.17, 14.74) | | 5.18 (0.00, 10.59) | | 2.06 (0.69, 3.44) |
| Urban or rural |  | |  | |  | |  |
| Urban area | 20.31 (14.36, 26.25) | | 21.46 (13.71, 29.21) | | 19.83 (13.48, 26.18) | | 19.36 (12.23, 26.49) |
| Rural area | 79.69 (73.75, 85.64) | | 78.54 (70.79, 86.29) | | 80.17 (73.82, 86.52) | | 80.64 (73.51, 87.77) |

**Table S5 Summary of results from the complete-case analysis for GHQ-12, SF-12 MCS, and SF-12 PCS**

| **Outcomes** | **Estimate (95% CI)** | **P-value** |
| --- | --- | --- |
| **GHQ-12** |  |  |
| Nurse (ref: Social interviewer) | -0.45 (-1.55, 0.65) | .98 |
| Web (ref: Nurse) | 0.63 (-0.68, 1.94) | .75 |
| Web (ref: Social interviewer) | 0.18 (-1.58, 1.94) | >.99 |
| **SF-12 MCS** |  |  |
| Nurse (ref: Social interviewer) | -0.89 (-2.84, 1.06) | .82 |
| Web (ref: Nurse) | 1.32 (-0.84, 3.49) | .43 |
| Web (ref: Social interviewer) | 0.43 (-2.59, 3.45) | >.99 |
| **SF-12 PCS** |  |  |
| Nurse (ref: Social interviewer) | -0.60 (-2.60, 1.41) | >.99 |
| Web (ref: Nurse) | -0.84 (-2.52, 0.84) | .70 |
| Web (ref: Social interviewer) | -1.43 (-3.68, 0.81) | .38 |

**Table S6 Summary of results from the complete-case analysis for cardiovascular conditions, diabetes, respiratory conditions, musculoskeletal disorders, and psychiatric conditions**

| **Outcomes** | **Odds ratio (95% CI)** | **P-value** |
| --- | --- | --- |
| **Cancer** |  |  |
| Nurse (ref: Social interviewer) | 1.06 (0.50, 2.22) | >.99 |
| Web (ref: Nurse) | 0.63 (0.24, 1.68) | .77 |
| Web (ref: Social interviewer) | 0.66 (0.22, 2.04) | >.99 |
| **Cardiovascular conditions** |  |  |
| Nurse (ref: Social interviewer) | 0.96 (0.59, 1.56) | >.99 |
| Web (ref: Nurse) | 0.62 (0.32, 1.20) | .25 |
| Web (ref: Social interviewer) | 0.59 (0.31, 1.14) | .16 |
| **Metabolic-endocrine conditions** |  |  |
| Nurse (ref: Social interviewer) | 0.94 (0.53, 1.68) | >.99 |
| Web (ref: Nurse) | 0.77 (0.40, 1.50) | >.99 |
| Web (ref: Social interviewer) | 0.72 (0.31, 1.67) | >.99 |
| **Musculoskeletal disorders** |  |  |
| Nurse (ref: Social interviewer) | 1.48 (0.84, 2.63) | .30 |
| Web (ref: Nurse) | 0.92 (0.54, 1.56) | >.99 |
| Web (ref: Social interviewer) | 1.36 (0.62, 2.98) | >.99 |
| **Psychiatric conditions** |  |  |
| Nurse (ref: Social interviewer) | 0.64 (0.34, 1.20) | .27 |
| Web (ref: Nurse) | 0.86 (0.35, 2.09) | >.99 |
| Web (ref: Social interviewer) | 0.55 (0.21, 1.44) | .41 |
| **Respiratory conditions** |  |  |
| Nurse (ref: Social interviewer) | 0.65 (0.36, 1.16) | .22 |
| Web (ref: Nurse) | 0.51 (0.26, 1.00) | .05 |
| Web (ref: Social interviewer) | 0.33 (0.14, 0.76) | **.005** |

**Table S7 Summary of results from the moderation analysis for GHQ-12, SF-12 MCS, and SF-12 PCS, stratified by sex**

| **Outcome** | **Group** | **Contrast** | **Estimate (95% CI)** | **Overall interaction P-value** | **P-value** |
| --- | --- | --- | --- | --- | --- |
| **GHQ-12** | Female | Nurse (ref: Social interviewer) | 0.61 (-0.98, 2.20) | .25 | >.99 |
|  |  | Web (ref: Nurse) | 0.09 (-1.61, 1.78) |  | .66 |
|  |  | Web (ref: Social interviewer) | 0.70 (-1.46, 2.85) |  | >.99 |
|  | Male | Nurse (ref: Social interviewer) | -1.22 (-2.68, 0.25) |  | >.99 |
|  |  | Web (ref: Nurse) | 0.43 (-1.71, 2.56) |  | >.99 |
|  |  | Web (ref: Social interviewer) | -0.79 (-3.40, 1.83) |  | >.99 |
| **SF-12 MCS** | Female | Nurse (ref: Social interviewer) | 0.60 (-1.83, 3.03) | >.99 | >.99 |
|  |  | Web (ref: Nurse) | 0.68 (-2.67, 4.02) |  | >.99 |
|  |  | Web (ref: Social interviewer) | 1.28 (-2.15, 4.71) |  | >.99 |
|  | Male | Nurse (ref: Social interviewer) | -1.39 (-4.36, 1.58) |  | >.99 |
|  |  | Web (ref: Nurse) | 0.01 (-2.77, 2.80) |  | >.99 |
|  |  | Web (ref: Social interviewer) | -1.38 (-5.36, 2.59) |  | >.99 |
| **SF-12 PCS** | Female | Nurse (ref: Social interviewer) | -0.80 (-3.84, 2.24) | >.99 | >.99 |
|  |  | Web (ref: Nurse) | -0.23 (-2.27, 1.81) |  | >.99 |
|  |  | Web (ref: Social interviewer) | -1.03 (-3.81, 1.75) |  | >.99 |
|  | Male | Nurse (ref: Social interviewer) | 0.13 (-2.21, 2.462) |  | >.99 |
|  |  | Web (ref: Nurse) | -1.08 (-3.45, 1.29) |  | >.99 |
|  |  | Web (ref: Social interviewer) | -0.95 (-3.69, 1.79) |  | >.99 |

**Table S8 Summary of results from the moderation analysis for GHQ-12, SF-12 MCS, and SF-12 PCS, stratified by age groups**

| **Outcome** | **Group** | **Contrast** | **Estimate (95% CI)** | **Overall interaction P-value** | **P-value** |
| --- | --- | --- | --- | --- | --- |
| **GHQ-12** | 16-25 | Nurse (ref: Social interviewer) | 0.66 (-4.75, 6.07) | >.99 | >.99 |
|  |  | Web (ref: Nurse) | -1.25 (-6.61, 4.12) |  | >.99 |
|  |  | Web (ref: Social interviewer) | -0.59 (-7.97, 6.80) |  | >.99 |
|  | 26-35 | Nurse (ref: Social interviewer) | 0.05 (-3.54, 3.64) |  | >.99 |
|  |  | Web (ref: Nurse) | -1.03 (-4.56, 2.51) |  | >.99 |
|  |  | Web (ref: Social interviewer) | -0.98 (-4.50, 2.55) |  | >.99 |
|  | 36-45 | Nurse (ref: Social interviewer) | -0.95 (-4.78, 2.89) |  | >.99 |
|  |  | Web (ref: Nurse) | 1.67 (-2.07, 5.41) |  | >.99 |
|  |  | Web (ref: Social interviewer) | 0.72 (-3.28, 4.73) |  | >.99 |
|  | 46-55 | Nurse (ref: Social interviewer) | 0.36 (-2.47, 3.19) |  | >.99 |
|  |  | Web (ref: Nurse) | 0.39 (-2.31, 3.09) |  | >.99 |
|  |  | Web (ref: Social interviewer) | 0.75 (-2.41, 3.91) |  | >.99 |
|  | 56-65 | Nurse (ref: Social interviewer) | 0.57 (-2.03, 3.18) |  | >.99 |
|  |  | Web (ref: Nurse) | 0.28 (-2.14, 2.70) |  | >.99 |
|  |  | Web (ref: Social interviewer) | 0.85 (-1.66, 3.37) |  | >.99 |
|  | ≥66 | Nurse (ref: Social interviewer) | -1.09 (-2.66, 0.47) |  | .66 |
|  |  | Web (ref: Nurse) | 1.08 (-1.15, 3.31) |  | >.99 |
|  |  | Web (ref: Social interviewer) | -0.01 (-2.46, 2.43) |  | >.99 |
| **SF-12 MCS** | 16-25 | Nurse (ref: Social interviewer) | 0.78 (-10.55, 12.10) | >.99 | >.99 |
|  |  | Web (ref: Nurse) | -4.42 (-11.78, 2.93) |  | >.99 |
|  |  | Web (ref: Social interviewer) | -3.65 (-14.27, 6.97) |  | >.99 |
|  | 26-35 | Nurse (ref: Social interviewer) | 1.26 (-6.08, 8.61) |  | >.99 |
|  |  | Web (ref: Nurse) | -2.54 (-9.39, 4.31) |  | >.99 |
|  |  | Web (ref: Social interviewer) | -1.27 (-6.81, 4.26) |  | >.99 |
|  | 36-45 | Nurse (ref: Social interviewer) | -1.41 (-7.10, 4.28) |  | >.99 |
|  |  | Web (ref: Nurse) | 3.69 (-2.56, 9.93) |  | >.99 |
|  |  | Web (ref: Social interviewer) | 2.28 (-4.25, 8.80) |  | >.99 |
|  | 46-55 | Nurse (ref: Social interviewer) | -0.73 (-5.79, 4.32) |  | >.99 |
|  |  | Web (ref: Nurse) | 1.56 (-3.61, 6.74) |  | >.99 |
|  |  | Web (ref: Social interviewer) | 0.83 (-5.00, 6.66) |  | >.99 |
|  | 56-65 | Nurse (ref: Social interviewer) | 1.35 (-3.79, 6.49) |  | >.99 |
|  |  | Web (ref: Nurse) | 0.77 (-3.47, 5.01) |  | >.99 |
|  |  | Web (ref: Social interviewer) | 2.12 (-2.03, 6.28) |  | >.99 |
|  | ≥66 | Nurse (ref: Social interviewer) | -1.62 (-4.84, 1.60) |  | >.99 |
|  |  | Web (ref: Nurse) | 2.08 (-2.60, 6.75) |  | >.99 |
|  |  | Web (ref: Social interviewer) | 0.46 (-4.76, 5.68) |  | >.99 |
| **SF-12 PCS** | 16-25 | Nurse (ref: Social interviewer) | -4.19 (-9.61, 1.22) | **.04** | .37 |
|  |  | Web (ref: Nurse) | -1.20 (-4.79, 2.39) |  | >.99 |
|  |  | Web (ref: Social interviewer) | -5.39 (-11.96, 1.18) |  | .25 |
|  | 26-35 | Nurse (ref: Social interviewer) | -0.23 (-4.74, 4.29) |  | >.99 |
|  |  | Web (ref: Nurse) | -0.57 (-4.54, 3.41) |  | >.99 |
|  |  | Web (ref: Social interviewer) | -0.79 (-4.34, 2.76) |  | >.99 |
|  | 36-45 | Nurse (ref: Social interviewer) | 1.90 (-4.90, 8.70) |  | >.99 |
|  |  | Web (ref: Nurse) | -1.31 (-6.99, 4.36) |  | >.99 |
|  |  | Web (ref: Social interviewer) | 0.59 (-4.19, 5.37) |  | >.99 |
|  | 46-55 | Nurse (ref: Social interviewer) | 2.53 (-2.15, 7.20) |  | >.99 |
|  |  | Web (ref: Nurse) | -2.27 (-6.64, 2.11) |  | >.99 |
|  |  | Web (ref: Social interviewer) | 0.26 (-4.43, 4.95) |  | >.99 |
|  | 56-65 | Nurse (ref: Social interviewer) | 0.43 (-4.73, 5.59) |  | >.99 |
|  |  | Web (ref: Nurse) | -2.08 (-7.03, 2.88) |  | >.99 |
|  |  | Web (ref: Social interviewer) | -1.65 (-5.99, 2.70) |  | >.99 |
|  | ≥66 | Nurse (ref: Social interviewer) | -2.37 (-6.82, 2.08) |  | >.99 |
|  |  | Web (ref: Nurse) | 2.48 (-2.76, 7.71) |  | >.99 |
|  |  | Web (ref: Social interviewer) | 0.11 (-5.17, 5.39) |  | >.99 |

**Table S9 Summary of results from the moderation analysis for cancer, cardiovascular conditions, metabolic-endocrine conditions, musculoskeletal disorders, psychiatric conditions, and respiratory conditions, stratified by sex**

| **Outcome** | **Sex** | **Contrast** | **Odds ratio (95% CI)** | **Overall interaction P-value** | **P-value** |
| --- | --- | --- | --- | --- | --- |
| **Cancer** | Female | Nurse (ref: Social interviewer) | 1.78 (0.63, 5.06) | >.99 | .86 |
|  |  | Web (ref: Nurse) | 0.51 (0.15, 1.69) |  | .83 |
|  |  | Web (ref: Social interviewer) | 0.91 (0.21, 3.86) |  | >.99 |
|  | Male | Nurse (ref: Social interviewer) | 0.80 (0.28, 2.31) |  | >.99 |
|  |  | Web (ref: Nurse) | 0.77 (0.19, 3.19) |  | >.99 |
|  |  | Web (ref: Social interviewer) | 0.62 (0.13, 2.89) |  | >.99 |
| **Cardiovascular conditions** | Female | Nurse (ref: Social interviewer) | 0.99 (0.52, 1.88) | >.99 | >.99 |
|  |  | Web (ref: Nurse) | 0.67 (0.29, 1.58) |  | >.99 |
|  |  | Web (ref: Social interviewer) | 0.66 (0.30, 1.49) |  | >.99 |
|  | Male | Nurse (ref: Social interviewer) | 1.13 (0.56, 2.28) |  | >.99 |
|  |  | Web (ref: Nurse) | 0.61 (0.30, 1.27) |  | .46 |
|  |  | Web (ref: Social interviewer) | 0.70 (0.30, 1.63) |  | >.99 |
| **Metabolic-endocrine conditions** | Female | Nurse (ref: Social interviewer) | 1.08 (0.48, 2.44) | >.99 | >.99 |
|  |  | Web (ref: Nurse) | 0.82 (0.32, 2.11) |  | >.99 |
|  |  | Web (ref: Social interviewer) | 0.88 (0.28, 2.73) |  | >.99 |
|  | Male | Nurse (ref: Social interviewer) | 0.79 (0.32, 1.98) |  | >.99 |
|  |  | Web (ref: Nurse) | 1.04 (0.39, 2.77) |  | >.99 |
|  |  | Web (ref: Social interviewer) | 0.82 (0.28, 2.41) |  | >.99 |
| **Musculoskeletal disorders** | Female | Nurse (ref: Social interviewer) | 1.15 (0.52, 2.54) | .25 | >.99 |
|  |  | Web (ref: Nurse) | 1.10 (0.51, 2.35) |  | >.99 |
|  |  | Web (ref: Social interviewer) | 1.26 (0.47, 3.41) |  | >.99 |
|  | Male | Nurse (ref: Social interviewer) | 2.43 (1.14, 5.20) |  | **.01** |
|  |  | Web (ref: Nurse) | 0.43 (0.19, 0.97) |  | **.04** |
|  |  | Web (ref: Social interviewer) | 1.05 (0.36, 3.04) |  | >.99 |
| **Psychiatric conditions** | Female | Nurse (ref: Social interviewer) | 0.94 (0.41, 2.17) | >.99 | >.99 |
|  |  | Web (ref: Nurse) | 0.49 (0.16, 1.50) |  | .56 |
|  |  | Web (ref: Social interviewer) | 0.46 (0.14, 1.56) |  | .57 |
|  | Male | Nurse (ref: Social interviewer) | 0.49 (0.18, 1.36) |  | .40 |
|  |  | Web (ref: Nurse) | 0.88 (0.21, 3.73) |  | >.99 |
|  |  | Web (ref: Social interviewer) | 0.43 (0.11, 1.66) |  | .60 |
| **Respiratory conditions** | Female | Nurse (ref: Social interviewer) | 0.82 (0.43, 1.59) | >.99 | >.99 |
|  |  | Web (ref: Nurse) | 0.64 (0.24, 1.69) |  | >.99 |
|  |  | Web (ref: Social interviewer) | 0.53 (0.17, 1.59) |  | .75 |
|  | Male | Nurse (ref: Social interviewer) | 0.71 (0.33, 1.56) |  | >.99 |
|  |  | Web (ref: Nurse) | 0.45 (0.20, 1.00) |  | .05 |
|  |  | Web (ref: Social interviewer) | 0.32 (0.10, 1.00) |  | **.049** |

**Table S10 Summary of results from the moderation analysis for cancer, cardiovascular conditions, metabolic-endocrine conditions, musculoskeletal disorders, psychiatric conditions, and respiratory conditions, stratified by age groups**

| **Outcome** | **Age** | **Contrast** | **Odds ratio (95% CI)** | **Overall interaction P-value** | **P-value** |
| --- | --- | --- | --- | --- | --- |
| **Cancer** | <56 | Nurse (ref: Social interviewer) | 0.91 (0.12, 6.97) | >.99 | >.99 |
|  |  | Web (ref: Nurse) | 0.65 (0.07, 6.07) |  | >.99 |
|  |  | Web (ref: Social interviewer) | 0.60 (0.06, 5.77) |  | >.99 |
|  | ≥56 | Nurse (ref: Social interviewer) | 1.29 (0.60, 2.76) |  | >.99 |
|  |  | Web (ref: Nurse) | 0.62 (0.19, 2.01) |  | >.99 |
|  |  | Web (ref: Social interviewer) | 0.80 (0.23, 2.83) |  | >.99 |
| **Cardiovascular conditions** | <56 | Nurse (ref: Social interviewer) | 0.93 (0.29, 2.96) | >.99 | >.99 |
|  |  | Web (ref: Nurse) | 0.64 (0.23, 1.81) |  | >.99 |
|  |  | Web (ref: Social interviewer) | 0.60 (0.20, 1.76) |  | >.99 |
|  | ≥56 | Nurse (ref: Social interviewer) | 1.10 (0.61, 1.99) |  | >.99 |
|  |  | Web (ref: Nurse) | 0.64 (0.29, 1.42) |  | .84 |
|  |  | Web (ref: Social interviewer) | 0.71 (0.33, 1.52) |  | >.99 |
| **Metabolic-endocrine conditions** | <56 | Nurse (ref: Social interviewer) | 0.82 (0.23, 2.97) | >.99 | >.99 |
|  |  | Web (ref: Nurse) | 1.40 (0.47, 4.19) |  | >.99 |
|  |  | Web (ref: Social interviewer) | 1.14 (0.32, 4.03) |  | >.99 |
|  | ≥56 | Nurse (ref: Social interviewer) | 1.02 (0.53, 1.94) |  | >.99 |
|  |  | Web (ref: Nurse) | 0.65 (0.30, 1.41) |  | .85 |
|  |  | Web (ref: Social interviewer) | 0.66 (0.25, 1.72) |  | >.99 |
| **Musculoskeletal disorders** | <56 | Nurse (ref: Social interviewer) | 1.89 (0.47, 7.58) | >.99 | >.99 |
|  |  | Web (ref: Nurse) | 0.56 (0.18, 1.73) |  | >.99 |
|  |  | Web (ref: Social interviewer) | 1.06 (0.27, 4.14) |  | >.99 |
|  | ≥56 | Nurse (ref: Social interviewer) | 1.45 (0.86, 2.45) |  | .38 |
|  |  | Web (ref: Nurse) | 0.84 (0.38, 1.90) |  | >.99 |
|  |  | Web (ref: Social interviewer) | 1.22 (0.47, 3.18) |  | >.99 |
| **Psychiatric conditions** | <56 | Nurse (ref: Social interviewer) | 0.70 (0.28, 1.78) | >.99 | >.99 |
|  |  | Web (ref: Nurse) | 0.67 (0.24, 1.93) |  | >.99 |
|  |  | Web (ref: Social interviewer) | 0.47 (0.15, 1.48) |  | .50 |
|  | ≥56 | Nurse (ref: Social interviewer) | 0.75 (0.30, 1.88) |  | >.99 |
|  |  | Web (ref: Nurse) | 0.48 (0.11, 2.09) |  | >.99 |
|  |  | Web (ref: Social interviewer) | 0.36 (0.09, 1.41) |  | .29 |
| **Respiratory conditions** | <56 | Nurse (ref: Social interviewer) | 0.76 (0.32, 1.78) | .86 | >.99 |
|  |  | Web (ref: Nurse) | 0.70 (0.29, 1.68) |  | >.99 |
|  |  | Web (ref: Social interviewer) | 0.53 (0.16, 1.70) |  | .89 |
|  | ≥56 | Nurse (ref: Social interviewer) | 0.79 (0.41, 1.53) |  | >.99 |
|  |  | Web (ref: Nurse) | 0.32 (0.13, 0.79) |  | **.005** |
|  |  | Web (ref: Social interviewer) | 0.25 (0.09, 0.71) |  | **.003** |

**Table S11 Supplementary analysis of the number of chronic-condition categories endorsed across survey modes in the imputed sample**

| **Contrast** | **IRR (95% CI)** | **P-value** |
| --- | --- | --- |
| Nurse (ref: Social interviewer) | 1.00 (0.83, 1.20) | >.99 |
| Web (ref: Nurse) | 0.72 (0.54, 0.96) | **.02** |
| Web (ref: Social interviewer) | 0.72 (0.50, 1.02) | .07 |

**Table S12 Summary of results from the main analyses for sensitivity analysis excluding participants affected by non-compliance for GHQ-12, SF-12 MCS, and SF-12 PCS, and chronic conditions**

| **Outcomes** | **Comparison** | **Effect measure** | **Estimate (95% CI)** | **P-value** |
| --- | --- | --- | --- | --- |
| **GHQ-12** | Nurse (ref: Social interviewer) | **β** | -0.29 (-1.30, 0.72) | >.99 |
|  | Web (ref: Nurse) |  | 0.40 (-0.85, 1.65) | >.99 |
|  | Web (ref: Social interviewer) |  | 0.11 (-1.48, 1.69) | >.99 |
| **SF-12 MCS** | Nurse (ref: Social interviewer) |  | -0.21 (-1.92, 1.50) | >.99 |
|  | Web (ref: Nurse) |  | 0.83 (-0.91, 2.57) | .76 |
|  | Web (ref: Social interviewer) |  | 0.62 (-1.59, 2.84) | >.99 |
| **SF-12 PCS** | Nurse (ref: Social interviewer) |  | -0.56 (-2.35, 1.24) | >.99 |
|  | Web (ref: Nurse) |  | -0.84 (-2.10, 0.42) | .33 |
|  | Web (ref: Social interviewer) |  | -1.40 (-2.97, 0.17) | .10 |
| **Cancer** | Nurse (ref: Social interviewer) | **Odds ratio** | 1.29 (0.63, 2.61) | >.99 |
|  | Web (ref: Nurse) |  | 0.53 (0.24, 1.19) | .18 |
|  | Web (ref: Social interviewer) |  | 0.68 (0.30, 1.56) | .81 |
| **Cardiovascular conditions** | Nurse (ref: Social interviewer) |  | 1.01 (0.66, 1.55) | >.99 |
|  | Web (ref: Nurse) |  | 0.77 (0.47, 1.25) | .58 |
|  | Web (ref: Social interviewer) |  | 0.78 (0.53, 1.14) | .35 |
| **Metabolic-endocrine conditions** | Nurse (ref: Social interviewer) |  | 0.96 (0.56, 1.64) | >.99 |
|  | Web (ref: Nurse) |  | 0.85 (0.47, 1.53) | >.99 |
|  | Web (ref: Social interviewer) |  | 0.81 (0.44, 1.50) | >.99 |
| **Musculoskeletal disorders** | Nurse (ref: Social interviewer) |  | 1.62 (0.97, 2.69) | .07 |
|  | Web (ref: Nurse) |  | 0.73 (0.47, 1.13) | .25 |
|  | Web (ref: Social interviewer) |  | 1.18 (0.67, 2.09) | >.99 |
| **Psychiatric conditions** | Nurse (ref: Social interviewer) |  | 0.79 (0.45, 1.40) | .97 |
|  | Web (ref: Nurse) |  | 0.75 (0.40, 1.40) | .82 |
|  | Web (ref: Social interviewer) |  | 0.60 (0.32, 1.11) | .14 |
| **Respiratory conditions** | Nurse (ref: Social interviewer) |  | 0.72 (0.43, 1.19) | .35 |
|  | Web (ref: Nurse) |  | 0.68 (0.38, 1.22) | .35 |
|  | Web (ref: Social interviewer) |  | 0.49 (0.26, 0.94) | **.03** |

**Figure S1 Adjusted risk differences (%) in reporting chronic conditions by survey mode, stratified by sex.**

**Figure S2 Adjusted risk differences (%) in reporting chronic conditions by survey mode, stratified by age groups.**
